# *“Everyone has their own problems and realities so the religious community cannot judge someone.”* Contraception motivations and perceived consequences among young contraceptive users who practice a religion in Burkina Faso

**DOI:** 10.1186/s40834-024-00295-7

**Published:** 2024-07-01

**Authors:** Fiacre Bazié, Ilene S. Speizer, Amelia Maytan-Joneydi, Kindo Boukary, Moh Fatimata Troaré, Balki Ibrahim Agali, Yentema Onadja, Georges Guiella

**Affiliations:** 1 Institut Supérieur des Sciences de la Population (ISSP), Université Joseph Ki-Zerbo, Ouagadougou, Burkina Faso Burkina Faso; 2 Department of Maternal and Child Health, University of North Carolina at Chapel Hill, Chapel Hill, NC USA; 3 Carolina Population Center, University of North Carolina at Chapel Hill, Chapel Hill, NC USA; 4 Groupe de Recherche et d’Action pour le Développement – GRADE Africa, Niamey, Niger

**Keywords:** Adolescent, Youth, Contraception, Religion, Burkina Faso

## Abstract

**Background:**

Numerous factors at the individual, interpersonal, and societal levels are related to contraceptive use (or non-use) among adolescents and youth. Little is known about the role of religion as an individual and community-level influencer of contraceptive use among young women.

**Methods:**

Using in-depth interviews with 24 young contraceptive users ages 18–24 who practice their Catholic, Protestant or Muslim religion in two cities in Burkina Faso, this study examines motivations and perceived consequences of contraceptive use. By including users of modern contraception who were both single and married, a range of perspectives are provided.

**Results:**

Generally, the young women interviewed who practice their religion and use contraception perceived that their religion was not supportive of contraceptive use. A few exceptions were provided, including perceived acceptance of use of some methods for married women for spacing purposes; this was specifically identified as acceptable among Muslim respondents. Sexual activity and contraceptive use were not acceptable by any of the religions for unmarried young women. That said, young women, both married and unmarried, reported their motivations for use that often related to their and their children’s health and the realities of life. Contraceptive use was considered a private matter which meant that the religious community would not find out about women’s use.

**Conclusions:**

Recognizing that some women are willing and able to use contraception even without the perceived support of their religious communities might help to push social norms to change and be more accepting of contraceptive use that meets young women’s and families’ circumstances.

## Background

Early and unintended pregnancy among adolescents (ages 10–19 years) and youth (ages 20–24 years) can have short- and long-term consequences on their own health and well-being as well as on the health and well-being of their children and families [1]. Access to and use of contraception for young people has become an important global goal to help improve young people’s lives and trajectories [2]. While contraceptive use has increased globally [3], increases have been unequal across geographies and by age groups, marital status, and parity. In particular, young people are often the least likely to use contraception and when they do use a method, they typically use the least effective methods [4]. Further, contraceptive use and use of the least effective methods are particularly problematic among unmarried young people [4]. In a recent multi-country study of young women ages 15–24 from sub-Saharan Africa, Behrman and colleagues [5] demonstrate distinctions in young women’s contraceptive use by region and birth experience. Their analysis shows higher contraceptive use among women with children from East/South Africa compared to women without children in that region; however, the opposite was true among women from West/Central Africa where women without children were more likely to use than women with any children [5]. Trends over time follow the same pattern and most of the use in both regions is of short-acting contraceptive methods such as oral contraceptive pills, male and female condoms, and injectables [5].

Numerous factors at the individual, interpersonal, and societal levels have been identified to be related to contraceptive use, non-use (or ineffective use) among adolescents and youth [6]. Among the societal factors, the influence of religious norms is important to highlight since these norms often support high fertility (and non-use of contraception) while at the same time they condemn sexual relations outside of marriage [7–10]. These religious norms are recognized as powerful barriers limiting access to and use of modern contraception, particularly among young women [7–11]. Social norms are perceived, unwritten rules that define acceptable and appropriate actions within a given group or community, thereby guiding individual and group behaviors [12–14]. According to theorists, there are two types of normative influences: a person’s belief about what other members of his or her group do (descriptive norms) and a person’s belief about what other members of the group approve and disapprove of (injunctive norms) [14–17]. Thus, descriptive norms influence health behaviors by providing information that people can use to guide their actions, while injunctive norms put pressure on people to meet the expectations of others [18–20]. In the specific area of ​​contraception, religious directives concerning fertility and sexuality are injunctive norms which limit access and use of contraception particularly among younger women. These norms, the sanctions that can result from their non-compliance, and self-sanctions that deter behaviors deemed deviant [21] are likely to have a significant influence on women’s reproductive behaviors.

To date, few studies have focused on the interpretation of these religious directives and their association with beliefs among young women who use modern contraception and who practice their religion, especially in the context of sub-Saharan Africa. However, understanding these perspectives would allow us to better understand how young women, living in a context of unfavorable norms, manage to overcome them to use contraception. This information can be useful for designing and implementing interventions aimed at countering the influence of so-called “harmful” norms on the promotion of universal access to reproductive health for all women, including young women.

Recent quantitative and qualitative studies have begun to examine the role of religion on contraceptive use behaviors among women, men, couples, and communities. For example, quantitative studies have demonstrated that there are distinctions in contraceptive adoption, method choice, and reproductive decision-making by religious affiliation [22–25]. These studies have found that Muslim women in union were significantly less likely to have ever used a contraceptive method than their non-Muslim counterparts [22, 23]; this has also been found in an analysis among young women ages 15–24 years [24]. Further, in an analysis of reproductive health decision-making, Darteh and colleagues [25] demonstrated that Muslim women were significantly less likely to make a decision about their reproductive health than their Christian counterparts.

Qualitative studies have also been undertaken to obtain a better understanding of how interpretations of religion in different contexts and communities are related to perceptions of contraceptive use and contraceptive decision-making. These studies have focused on women of reproductive age [26–30], men of reproductive age [26, 28–30], providers [31], and religious leaders [28, 31–35]. A notable gap in qualitative (and quantitative) studies on religion and its association with contraceptive use has been an examination of how religion and interpretations of religious doctrines by women, adolescents, and religious leaders are associated with young people’s (i.e., under age 25) and unmarried youth’s contraceptive decision-making and their access to and use of sexual and reproductive health (SRH) information and services. This qualitative study begins to fill this gap and focuses on young women who use contraception to understand their perspectives of their religion’s acceptability of contraceptive use in two cities of Burkina Faso. It goes on to examine among young female users of contraception, their perspective on how religion influences their contraceptive decision-making processes and the perceived consequences of their current contraceptive use.

## Methods

### Study context

This qualitative study took place in Ouagadougou and Bobo Dioulasso, the capital city and the second largest city, in Burkina Faso. Burkina Faso is a land-locked country in francophone West Africa with an estimated 2021 population of 22 million.^1^ In 2020, about 43% of the population is estimated to be under age 15 years of age^2^ and 20% of the female population is estimated to be in the ages 15–24.^3^ In 2021, it was estimated that about 61% of the population of Burkina Faso was Muslim, 19% were Roman Catholic, 4% were Protestant, and about 15% practice another indigenous religion [36]. Ouagadougou is in the Centre region of the country; the religious distribution in Ouagadougou is 58% Muslim, 33% Catholic, 8% Protestant, and the remaining reporting no religion or another religion. In Bobo Dioulasso, the population is more concentrated in the Muslim faith with 78% Muslim, 17% Catholic, and 4% Protestant.^4^

In Burkina Faso, in 2021, 30% of women of reproductive age were using any method of contraception; the overwhelming majority of this use (29%) is of modern methods including pills, condoms, injectable, IUD, implant, lactational amenorrhea, male and female sterilization, Standard Days Method, and emergency contraception. Among users of any method, 43% are using implants with injections being the next most common method at 22%. Among women ages 15–19, only 13% were using a method (12% modern methods) whereas among women ages 20–24, 35% are currently using a method (32% modern). Implants remain the most commonly used method (38% of method use) among women ages 20–24, whereas among women ages 15–19, condoms (42%) are the most commonly used method followed by implants (25%). Comparing by city, a greater percentage of 15–19 year olds from Bobo Dioulasso (13%) use a modern method than their counterparts in Ouagadougou (8%); this difference was not significant. Comparing differences among 20–24 year olds, we see again that a greater percentage of those from Bobo Dioulasso are using a modern method (46%) compared to their counterparts from Ouagadougou (33%); this difference was significant at *p* = 0.028.^5^

### Study sample and approach

In each study site, qualitative data were collected from young women and religious leaders. This study focuses on the data from in-depth interviews with 24 young women ages 18–24 across the two cities.^6^ Given that the focus of the study was to understand the role of religion and religious leaders on young women’s contraceptive decision-making and use, we included a select sample of young women ages 18–24 who were using contraception. Since use among younger women (e.g., ages 15–17) is less common, it was determined that this slightly older sample will better reflect the perspectives of young users. Selection criteria included that they were current users of a modern method of contraception including intrauterine device, implant, injectables, pills, male or female condoms, and emergency contraception. The other important selection criterion was that participating young women had to report that they practice their religion. If the young person was Muslim, she had to report that she prays five times a day and participates in activities at her mosque or with her religious community. If the young woman was Christian (Catholic or Protestant), she needed to report that she goes to church at least once a week and participates in activities within her religious community outside of church services. A screening tool was used to ask young women identified in the recruitment phase (see below) if they met these contraceptive use and religious practice criteria. Quota sampling was used to ensure that we included an appropriate number of contraceptive users from the different religion and marital status groups (see Table 1).

**Table 1:** Characteristics of young contraceptive users interviewed in Ouagadougou and Bobo Dioulasso, Burkina Faso

Characteristic	Bobo Dioulasso	Ouagadougou	Total
Age			
18–19 20–22 23–24	0 3 9	2 2 8	2 5 17
Marital status			
Never married Married/in union/ever married	6 6	6 6	12 12
Religion			
Catholic Protestant Muslim	4 4 4	4 4 4	8 8 8
Current method use			
IUD Injectable Implant Pill Condom	1 2 6 3 0	0 4 2 2 4	1 6 8 5 4

Recruitment happened with the assistance of health workers in the study communities since we were seeking to interview users of contraception. Female community health workers approached young women who they knew were users of contraception and asked them to meet with interviewers for screening purposes. All screening happened by study interviewers who identified potential participants’ contraceptive use, religion, and marital status to ensure a mix of users of different religions and marital statuses. Among users of contraception, we included married and unmarried young women from each of the two study sites. Once an eligible young woman was identified, she was read the consent form in the local language and asked to give written consent to participate. Those who consented were interviewed in a private location by a trained female interviewer who spoke the local language. Interviews took about an hour on average. Those who chose not to participate or were not eligible were thanked and given a beverage. Following completion of the interview, participating young women were also given a beverage.

A semi-structured interview guide was developed that asked questions about the woman’s contraceptive experience, influencers of contraceptive use, perceptions of her religion’s acceptance of contraception, and how she views her religion and contraceptive decision-making. This was meant to capture the role of injunctive and descriptive norms on young women’s contraceptive decision-making and behaviors. Interviews were undertaken in French or in one of the local languages of the two study cities (Dioula and Mooré). All recruitment materials, consent forms, and semi-structured interview guides were reviewed and approved by the Comité d’Ethique pour la Recherche en Santé in Burkina Faso (#2022-06-122) and by the Institutional Review Board at the University of North Carolina at Chapel Hill (#22-1125).

Interviews were recorded, transcribed, and translated into French for coding and analysis. The study team used Dedoose, a collaborative qualitative data software. As a first step, five team members separately coded one transcript using an initial codebook developed based on the interview guide and then the team met to compare coding. This permitted identification of new parent and child codes for a more comprehensive coding process and alignment of the coding team. A second transcript was then separately coded and alignment across coders discussed. Once this process was completed, each transcript was coded by one member of the team using the finalized codebook. Once all data were coded, a thematic analysis was undertaken to identify key themes in the data based on the main codes that emerged from the interviews. Matrices were created that assessed the depth related to specific points being made by study participants. Below we present the results by key themes identified. All citations included in this paper were translated into English and then rechecked by the Burkina Faso team to ensure that they reflected the underlying meaning of the quote. It is worth noting that in the local languages the terms for family planning, contraception, and birth spacing are all synonymous. This means that when the translation happened into French, all of these terms are used, depending on the understanding of the translator. Here the English translations use the same word that was captured in French to remain as close to the respondent’s meaning as possible.

## Results

Table 1 presents the characteristics of the young women interviewed in Bobo Dioulasso and Ouagadougou. As can be seen in the table, the majority of the women were in the oldest ages (age 23–24 years). Half of the sample were never married while the other half were ever married or currently in union. The sample is evenly split across the religious groups in both cities. That said, the current modern method used in the two cities varies with more implant users interviewed in Bobo Dioulasso and more injectable and condom users interviewed in Ouagadougou.

Findings from the in-depth interviews were examined under several key themes. These were: (a) perspective of religion’s acceptability of contraceptive use; (b) reasons and motivations for using contraception under perceived unsupportive religious circumstances; and (c) perceived consequences from one’s religious community for using contraception. It is worth noting that when the adolescents and youth were talking about their perspectives and experiences, they were often referring to two types of scenarios – married users, no matter the age; and unmarried users, often equated with questions around adolescent and youth use.

### Perspectives on religion’s position on contraceptive use

During the interviews, the young modern method users were asked about their knowledge of their respective religion’s position on contraception. In general, all respondents had an understanding of the position of their religion on contraception. This knowledge was acquired either through direct contact with religious leaders or from their sermons on the topic. Others learned about their religion’s views on contraceptive use from discussions within their family and in some cases through personal readings of religious texts. Across all religions, generally the respondents felt that their respective religions are opposed to the use of contraception or any means to avoid becoming pregnant, no matter the age or marital status of the person using. For example, these two married young women talked about religious prohibitions against contraceptive use.

*In the sermons they often say that it is forbidden to use contraceptive methods because it is a sin to do so.*

*Married Muslim Woman from Ouagadougou*

*Religion is against the use of contraceptive methods; whether it is the pill or the other methods, religion is against it.*

*Married Catholic Woman from Bobo Dioulasso*

According to the young users, the prohibition of contraceptive use is based on religion’s perspective that a woman or couple should not limit the number of children they have. The child is considered “a gift from God” and the different holy scriptures (the Bible and Koran) as well as the religious communities across the religions have a strict position on this subject. Avoiding getting pregnant is therefore contrary to the will of the three religions included and therefore perceived as a sin.

*In the church it is said during the prayers that the woman must never limit, nor space her births, the number of children that God gives you only you must give birth to them. It has never been written in the Bible that a woman can do family planning. Rather, it is written that the woman must have as many children as God will give her.*

*Unmarried Catholic Woman from Bobo Dioulasso*

*My husband himself is a practicing Muslim who has finished reading the Koran and who teaches the Koran to people, it was he who always told me that it is forbidden in Islam to use contraception. He told me never to use contraception, that the number of children that God has allowed all Muslim women must do and never seek to limit births. He also added that a child is a gift from God, it is God who gives and it is he who takes care of it and no one else ah (smile).*

*Married Muslim Woman from Ouagadougou*

In the same sense, several respondents felt that their religion considers that the use of contraception to avoid becoming pregnant amounts to “terminating the life of a child” and this is considered a serious sin.

*It is when we pray in church, we often open parentheses to say that if you do contraception, after death you will go directly to hell insofar as you kill children. That if God had planned five children for you and if you practice contraception for five before your marriage, it is your children that you are killing. Now after your marriage you want children when you have already killed them.*

*Unmarried Catholic Woman from Ouagadougou*

Importantly, according to many of the respondents, the debate on the position of religion and contraceptive use is only relevant for women in union. Concerning single people, sex before or outside marriage is strictly prohibited and thus they should not be part of the discussion of religion and contraceptive use.

*In the Muslim religion it is formally forbidden for the young unmarried woman to have intimate relations with a man outside marriage. In any case, the religious leaders talk about it and they advise young single women to get married before having intimate relations with a man. At this time they do not even talk about methods concerning them.*

*Married Muslim Woman from Ouagadougou*

### Nuanced views about religion and contraceptive use based on type of method and marital status

While overall, most respondents feel that their religion prohibits contraceptive use, there were some respondents who talked about ambiguities of religious doctrines and leaders’ statements about family size and family planning (FP).

*No, it’s general. For them, they are against everything that prevents the birth of a child. But they also say not to make children more than our ability to care for them. Now how are you going to make sure a child doesn’t come? We want something and its opposite? It is complicated.*

*Married Catholic Woman from Ouagadougou*

Some women acknowledged that some methods of contraception were more acceptable, and that use was considered appropriate for some married women. In particular, across the religions, some respondents felt that the use of traditional methods (i.e., withdrawal or rhythm method) is authorized for spacing births. In addition to these traditional methods, the condom was considered acceptable by some of the respondents across the religions.

*What is allowed in the Muslim religion on FP is above all the use of methods such as condoms, the rhythm method or women who are educated calculate their cycle, there is also the method coitus interruptus. These are really methods that we do not say that the religion has forbidden because that is what many married Muslim women use.*

*Unmarried Muslim Woman from Ouagadougou*

To a lesser extent, some young women felt that the use of FP from a religious point of view was accepted provided that the woman had the prior consent of her husband/partner.

*As far as married women are concerned, we always say that your use of FP depends on your husband, if he wants you to have a child, that’s it, if he wants that much, he’s the one who decides.*

*Unmarried Protestant Woman from Ouagadougou*

### Reasons and motivations for using contraception under unsupportive religious circumstances

Despite perceived religious injunctions on the use of FP and the awareness they have on this subject, the young women interviewed noted their reasons or motivations for their contraceptive use. The reasons or motivations given were diverse but similar across the different religions and can be distinguished according to the marital status of the young women. The majority of young women in union mentioned as the first reason for using contraception the difficulties inherent in contemporary life, in particular financial difficulties in coping with the care of the family. This imposes the need to space children and sometimes limit births.

*I say we have no choice, it’s because of real life. I told you what I do for work; my husband is also a trader and the market is not stable. If you have a child in school and other children in the private sector, will you be able to get by? It’s difficult. Religion also says that abortion is not a good thing and in order not to condemn you with God, it is better to take the path where you can justify yourself later.*

*Married Catholic Woman from Ouagadougou*

*Nowadays life has become very hard. For example, if you do not use contraceptive methods and each time you give birth to children and the spacing between them does not exceed a year like that and if your husband does not have enough means it is difficult. Also, the children have to attend [school], you have to feed them, take care of them so when there are many of them and then there are not enough means there it is difficult; this is the reason why I decided to use the contraceptive method. I use FP I know it’s not good in the Muslim religion but I hope God will forgive me one day.*

*Married Muslim Woman from Ouagadougou*

The young married women also mentioned reasons for use related to the health of the mother and the child.

*It is true that our religion does not support the use of contraception, may God only forgive us because I thought about my health before making the decision to do so. I thought about my child’s health before I did. I saw that if I space my births well I can take good care of them.*

*Married Muslim Woman from Ouagadougou*

For young married women, they bear the brunt of the consequences and burdens associated with pregnancy and childcare. For this, they use contraception for their own well-being despite the religious discourses that oppose this.

*Other men refuse, but it’s up to you, the woman, to think about it because the man doesn’t care, it’s you who will be there with very young children lined up and you can’t do anything. He will get up to go about his business and leave you with your children. For this, even if no one tells you, you must try to do FP unless you yourself are insane.*

*Married Catholic Woman form Ouagadougou*

Concerning young unmarried women, the main reason cited for use is related to social, family, and religious sanctions that can result from pregnancy before marriage in a context where cultural and religious norms emphasize abstinence until marriage, a practice that is increasingly difficult for girls to respect. Pregnancy before marriage is considered a dishonor for the family and the consequences include stigmatization of all kinds and in some cases the repudiation or expulsion of the young woman from the family circle. Thus, the use of contraceptive methods is perceived as a means for unmarried young people to be sexually active but also to protect themselves from the sanctions and other consequences which could result from an unwanted or extramarital pregnancy.

*Ah if you see that I myself put religion aside and went to do it, it’s because it’s personal. I went to do… it’s getting pregnant, that’s the problem. You see in family, especially us the Mossis [ethnic group] even, we are complicated. If a child (a girl) becomes pregnant, she is divorced from the family, as long as they do not do certain things to fix it (tradition), you cannot return (to the family); so I went to do so as not to get pregnant; I want to honor my parents and those from where I go to pray.*

*Unmarried Muslim Woman from Bobo Dioulasso*

*That’s what I said, it’s because of what our community members say, because if you have a child before marriage, we’ll insult you that you got pregnant while you’re going to the church.*

*Unmarried Catholic Woman from Bobo Dioulasso*

Overall, for these young, unmarried religious women, the reasons for violating religious prescriptions by using FP are all social and cultural. While young brides use contraception mainly to space births, single people use it to delay the onset of fertility, which is poorly accepted if it happens outside marriage. Sanctions and stigmatization of all kinds constitute the main fear pushing young unmarried women to use FP.

### Use of contraception and religious practice

The young respondents were asked directly about their use of contraception and how this relates to their religious beliefs and practices. Some of the respondents reported that their contraceptive use does not affect their religious practices or beliefs, that is, they were able to separate these issues.

*Because using contraception has never stopped me from always practicing my religion, it doesn’t stop me at all. Whether I use FP or not, it’s the same thing, I practice my religion without any problem. When I see my period only I cannot pray for four days and that is long before I start using the contraceptive method. So for me, I don’t see any negative impact of contraception on religious practice or belief.*

*Unmarried Muslim Woman from Bobo Dioulasso*

*Because we do, but while continuing to practice our religion. FP is like an aid that allows us to space births; it’s like they say help yourself and God will help you too. One does not prevent the other.*

*Married Catholic Woman from Bobo Dioulasso*

Some of the young women did struggle with their contraceptive use and how this affects their religious practices and beliefs. The messages from their religion are important in affecting their decision-making to use or continue contraceptive use. This unmarried Catholic woman from Ouagadougou expresses these concerns:

*I think that what I did is not good because it is a life that I eliminate so I think my faith is a little reduced, it is not complete insofar as my use of FP kills children and I think that after this dose I will stop contraception because I am killing children. (….) Because we were told in church that we kill children when we use FP. That we are reducing the number of children that God has given us.*

### Perceived consequences from religious community for using contraception

During the interviews, respondents were asked what were the perceived consequences or sanctions a young woman could face if her religious community found out that she was using contraception despite perceived religious prescriptions prohibiting its use. On this subject, some respondents, mostly single Christian women, mentioned consequences from the religious community, such as the denial and withdrawal of certain responsibilities that formerly fell to the young women in question and even a ban on frequenting the church.

*Well, for example, if I had responsibilities in the church, they can remove me from my duties, I would no longer be responsible for these responsibilities. They can decide to punish you, but I don’t know but they can even tell you not to come to church for a long time, somehow they punish you.*

*Unmarried Protestant Woman from Ouagadougou*

*With us, if you are a young girl and you do the work of God, for example if you sing or if you perform, they will forbid you all that. You won’t be able to do anything for God yet. They’re going to write it down on a paper and read it before the whole church.*

*Unmarried Protestant Woman from Bobo Dioulasso*

Notably, for the majority of respondents across the religions, the expectations of sanctions because of their contraceptive use were rare with the women acknowledging that it is difficult for their families and religious community to know that a woman is using contraception because use is systematically done in a hidden way. For them, the possible consequences that could result from the discovery of contraceptive use would mainly be counseling by religious leaders and also possibly community member stigmatization.

*Ah, they will take it badly, it will not be good for me and the reputation of my family who attend the mosque. But it will never happen because I don’t see how they will find out that I use.*

*Married Muslim Woman from Ouagadougou*

Others, on the other hand, consider that there is no need to fear possible negative consequences on the part of the religious community.

*Like for example telling me not to come to the choir anymore? Or refusing to allow me to participate in church activities? No no !! They’re not going to do that, it’s not going to get to that level, they’re just going to advise you.*

*Unmarried Catholic Woman from Bobo Dioulasso*

On the contrary, for some young women (mostly Christian) they did not foresee consequences from their religious community. In the end, these women report that the decision to use or not to use contraception remains an individual and private choice, a choice that the young woman or the couple make according to their realities and their well-being.

*There is nothing that can happen to her because the others do not know what the person lives to judge her. Since the community doesn’t know why you did the FP, it can’t do anything to you. The use of FP can be a relief for the person; it is a responsibility that only engages the person so the community cannot say anything.*

*Unmarried Catholic Woman from Ouagadougou*

*Since it’s our life for both of us, my husband and I, we decide to do so. We are both Protestants and so we follow each other to seek paradise so it’s like that and it’s us who know how to do so that the family is in peace and joy.*

*Married Protestant Woman from Ouagadougou*

Ultimately, according to the women, only God is able to pass judgment on their actions and not the religious community.

*The religious community cannot judge people in place of God, that is not their task, it is God alone who judges. Everyone has their own problems and realities so the religious community cannot judge someone.*

*Unmarried Muslim Woman from Ouagadougou*

*No, I don’t mind my faith. It is in God that I believe, it is only he who can judge me. A human being cannot judge me. The fact that God does not test me because I use birth control does not bother me.*

*Unmarried Protestant Woman from Bobo Dioulasso*

Overall, although some young women raised fears of sanctions from their religious community regarding the use of FP, for the majority, the consequences of this use can be summed up only in warnings, advice or limited stigmatization. However, the risks of sanctions, even if they exist, are not likely to curb their desire to use contraception, which for them responds to their current social and economic realities. Moreover, they believe that the use of FP remains a private matter that concerns only the young woman (and her husband/partner) and that only God can judge.

## Discussion

This qualitative study from two cities in Burkina Faso among young contraceptive users who practice their Muslim, Catholic, or Protestant religion showed that there is a common perspective that religion generally prohibits contraceptive use (see Table 2). On the one hand, there is the perspective that all religions condemn the use of contraception without restrictions; however, there were exceptions made for spacing and traditional method use across the religions and for married women. There is also a general agreement that all religions condemn sexual intercourse among single people.

**Table 2:** Summary of young contraceptive users’ perceptions, motivations and consequences related to their religion and contraceptive use by religion and marital status

Religion	Marital status	Perception of religion/religious positions	Personal motivations to use	Perceived consequences of use
Muslim	Married	- Opposition for use and sometimes favorable for use: o Traditional methods o For spacing o Under partner approval	- For spacing childbirth - To avoid (financial) difficulties of modern life - For mother and child health/well being	- No consequences - May be advised
	Unmarried	- Opposition for use no matter type of method	- To delay first birth - To avoid sanctions and stigma related to an unwanted or extramarital pregnancy	- No consequences - May be advised
Christian (Catholics and Protestants)	Married	- Opposition for use and sometimes favorable for use: o Traditional methods o For spacing o Under partner approval	- For spacing childbirth - To avoid (financial) difficulties of modern life - For mother and child health/well being	- No consequences - May be advised
	Unmarried	- Opposition for use no matter type of method	- To delay childbirth - To avoid sanctions and stigma related to an unwanted or extramarital pregnancy	- No consequences - May be advised - Fear of denial and withdrawal of certain responsibilities

Among those who discussed acceptable contraceptive use practices, it was felt that use was essentially for spacing and not limiting births (see Table 2). The women, especially the Muslim women, acknowledged that spacing births up to two years through breastfeeding or another means was considered acceptable, even if they did not specifically say that the religion permitted modern contraceptive use to meet this objective. These perspectives go against the perceived prohibitions against contraceptive use but demonstrate the importance to these young women of doing what they felt was better for their health and the health of their children.

The results from this study are interesting when compared to a global review of religion and contraceptive use. Pinter and colleagues [37] demonstrate that contraception is generally acceptable for married women among Protestants and acceptable to support the health of married women and their children among Muslims. That said, the Catholic religion does not consider any modern methods of contraception acceptable. Our findings illustrate that all participants perceived that their religion was not supportive; however, they used a contraceptive method nevertheless. This was true across the religious groups as well as marital status groups. Of course, this study specifically enrolled users and there are likely many non-users who are influenced by these same beliefs and are not using a method.

Prior quantitative studies typically show greater contraceptive use among Christian (Protestant and Catholic) women as compared to Muslim women [22, 38]. This may reflect greater autonomy of Christian women to go against their religious beliefs and use a contraceptive method when or if they need to. In this study, we find that young users (married and unmarried) are making conscious decisions to use based on their own and their families’ needs. When examining the perceived sanctions for their use, we saw that some of the Christian women felt that the consequences of their use would be minimal in their religious community and for the most part, their religious community was unlikely to learn of their use. These young Christian women likely have greater decision-making autonomy which is consistent with the study by Darteh and colleagues [25] that found that Christian women were significantly more likely to be part of reproductive health decisions than their Muslim counterparts. To explore this in more depth requires a broader sample of young users and non-users from the different religious groups.

Among young married women, the husband’s consent and support was perceived as a sort of override on the religious prohibitions. This acceptance may reflect the effect of gender norms that encourage women to follow the will of their spouse, who is supposed to know religion better than them, rather than specifically focusing on a religious doctrine. This highlights gender norms that typically place the responsibility for daily family needs on the women but the decision-making around FP and family size on men [39]. In a recent study from Burkina Faso, men are a key barrier to women’s FP use, not only because of their desire for large families but also due to their lack of knowledge about FP, negative beliefs and perceptions about contraception, and a lack of engagement in FP programming [30].

Despite the perception among women of the different religions that contraceptive use is not allowed, these young women who practiced their religion still opted for the use of contraceptive methods for reasons they consider more urgent and important than compliance with perceived religious guidelines. The motivations to use contraception were essentially linked to the young women’s socioeconomic and cultural realities, as found previously [39]. FP use among women of all ages and religions is often undertaken for the betterment of women, couples, and families, no matter the perceived community norms surrounding contraceptive use. Here, young women, despite the practice of their religion and the strong perspective of unfavorable religious norms, make choices that address the various difficulties imposed on them.

These results, which ultimately show a limited influence of religious restrictions on young women’s contraceptive behaviors, despite the fact that they still constitute norms widely shared by young women, offer us interesting interpretations in connection with the different theories linked to the influence of social norms on human behavior. Indeed, if community norms, through prescriptions, proscriptions and social sanctions, affect individuals’ behaviors, the different theories suggest that this influence may not be systematic and human behavior may be more influenced by other factors, particularly individual factors [19, 40–45]. Among these factors, individual expectations are important, particularly the advantages perceived by adopting the behavior [19, 45] and attitudes related to the behavior (favorable or unfavorable) [41, 46]. In our present case, the motivations of young women to use contraception are mainly linked to the socio-economic and health advantages and benefits that they anticipate through this use. These perceived advantages can also be seen as individual attitudes [41, 44] which are favorable to the use of contraception. Our results show that it is really these perceived advantages (or individual attitudes) which influence the individual behaviors of young women and much less the norms or religious directives from their community.

On the other hand, research also suggests that normative influence also depends on certain attributes of behavior, notably its public or private character. Indeed, according to theorists, the extent to which a behavior is adopted in a public or private settings is likely to moderate or accentuate the influence of the social norm [19, 44, 47]. As Rimal and his colleagues [44] conceived, if a behavior is adopted in a private setting, the pressures exerted to conform to the behaviors of others or to their beliefs about the course of action to adopt (injunctive norms) would be less relevant, particularly because the behavior is not observed by others and is therefore less likely to be subject to potential social sanctions. In contrast, for behaviors that are engaged in a public setting, knowing that one’s behaviors can be scrutinized by the public implies that social sanctions can be exercised in the event of violation of injunctive norms. Under these conditions, the pressures exerted to conform, that is to say, to adopt behaviors perceived as acceptable in the eyes of others, are likely to be significant. Here, we can consider that areas of sexuality including the use of contraception fall within the private domain where the community and the (extended) family have relatively little influence, especially in the urban context. Social and religious sanctions, in the event of violation of norms, which should ultimately contribute to producing normative behavior, are then unlikely to be exercised. The private nature of sexuality, including the use of contraception, could justify the moderate fear of sanctions that we observe, particularly among young single women.

### Limitations

This study that included young contraceptive users practicing their religion has some potential limitations. First, given the sensitivity of the subject, it is possible that the respondents who agreed to take part in the study could be young women who are more open and motivated to the idea of FP and less under the influence of religious constraints. Second, these results conceal the case of young religious women who do not have access to contraception because of these same norms. This would attenuate the importance of religion in the study sample. Third, because this is a qualitative study it is not generalizable beyond the study sample and study sites.

Despite these limitations, this study provides knowledge on the articulation between religious beliefs and the use of FP among adolescent girls and young women in Burkina Faso. On the one hand, it highlights the understanding and interpretation of the three religion’s positions on FP use and, on the other hand, it shows how young religious women rationalize their choice to use contraception despite perceived unfavorable religious prescriptions.

## Conclusions

Torn between respect for norms and religious prohibitions concerning contraception and the realities of contemporary life, young married and unmarried women often decide to use contraceptive methods in a hidden way. Covert use is necessary because it allows the young women to meet their reproductive goals in accordance with their current realities. However, the majority of young women in Burkina Faso and elsewhere, remain under the influence of various constraints, including religion, preventing them from having access to or using contraceptive methods and from satisfying their reproductive health needs and rights. Recognizing that some women are willing and able to use contraception even without the perceived support of their religious communities might help to push social norms to change and be more accepting of contraceptive use that meets women and families’ personal and financial circumstances. Better targeted communication towards the different religious communities could lead to a change in perspectives of family planning and its comparative advantages for women and for families in general.

Further, future initiatives that provide young people with accurate information and resources on sexual and reproductive health are important to counter misinformation or address their perceptions that their religious communities are not supportive of contraceptive use. This might include strengthening family life education in schools; these types of programs aim to strengthen young peoples’ sexual and reproductive health knowledge and life skills. These types of initiatives, if well implemented, that is with fidelity and including comprehensive information including the benefits of using contraception to delay and space births, can equip young people with favorable attitudes and skills to counter the effects of harmful norms and allow young people to enjoy their reproductive rights.

Finally, this study that used qualitative data, made it possible to explore the complexity of the relationships between religious norms and individual behaviors, allowing us to understand how individual motivations often surpass the strength of social norms in areas recognized as sensitive such as that of religion and its links to contraceptive use. This once again indicates the relevance of qualitative data if we want to better understand the explanations underlying individual experiences, particularly on sensitive subjects.

## Data Availability

The qualitative data generated and analyzed during the current study are not publicly available in order to protect the identities of the participants involved but are available from the corresponding author (speizer@email.unc.edu) on reasonable request that clarifies how the data will be used and provides plans for safeguarding the data in a manner that protects the participantsidentities.

## Footnotes

1 https://data.worldbank.org/indicator/SP.POP.TOTL?locations=BF.

2 https://www.unfpa.org/data/world-population/BF.

3 https://www.unfpa.org/data/adolescent-youth/BF .

4 Calculated from the 2021 Burkina Faso Demographic and Health Survey raw data.

5 Based on calculations using the 2021 Burkina Faso Demographic and Health Survey.

6 Information from the religious leaders and focus group discussions with young users are presented in a separate analysis.

## Abbreviations

FP: Family planning
SRH: Sexual and reproductive health
